# A polymer‐based systemic hemostat for managing uncontrolled bleeding

**DOI:** 10.1002/btm2.10516

**Published:** 2023-04-13

**Authors:** Yongsheng Gao, Mayumi Ikeda‐Imafuku, Zongmin Zhao, Maithili Joshi, Samir Mitragotri

**Affiliations:** ^1^ John A. Paulson School of Engineering and Applied Sciences, Harvard University Allston Massachusetts 02134 USA; ^2^ Wyss Institute for Biologically Inspired Engineering at Harvard University Boston Massachusetts 02115 USA

**Keywords:** hemophilia, hemostasis, hyaluronic acid, systemic hemostat, traumatic bleeding

## Abstract

Uncontrolled bleeding is a life‐threatening emergency that requires immediate intervention. Currently available on‐site bleeding interventions largely rely on the use of tourniquets, pressure dressing, and other topical hemostatic agents, which can only treat bleeding injuries that are known, accessible, and potentially compressible. Synthetic hemostats that are stable at room temperature, easy to carry, field‐usable, and able to stop internal bleeding at multiple or unknown sources, are still lacking. We recently developed a hemostatic agent via polymer peptide interfusion (HAPPI), which can selectively bind to activated platelets and injury sites after intravascular administration. Here we report that HAPPI is highly effective in treating multiple lethal traumatic bleeding conditions in normal as well as hemophilia models via either systemic administration or topical application. In a rat liver traumatic model, intravenous injection of HAPPI resulted in a significant decrease in blood loss and a four‐fold reduction in mortality rate within 2 h after injury. When applied topically on liver punch biopsy wounds in heparinized rats, HAPPI achieved a 73% of reduction in blood loss and a five‐fold increase in survival rate. HAPPI also exhibited hemostatic efficacy in hemophilia A mice by reducing blood loss. Further, HAPPI worked synergistically with rFVIIa to induce immediate hemostasis and 95% reduction in total blood loss compared to the saline‐treated group in hemophelia mice models. These results demonstrate that HAPPI is a promising field‐usable hemostatic agent for a broad range of different hemorrhagic conditions.

## 1 INTRODUCTION

Uncontrolled bleeding is one of the most common causes of death, contributing to approximately 1.9 million worldwide deaths annually.^1^ Excessive hemorrhage in trauma patients or patients with bleeding disorders is particularly life‐threatening. It is estimated that 30%–40% of all trauma‐related deaths^2^, ^3^, ^4^, ^5^ and 20%–30% of deaths among hemophiliacs^6^, ^7^, ^8^ result from hemorrhage. Because severe bleeding is a time‐sensitive condition with a median time to death of 2 h,^9^ a majority of hemorrhagic deaths occur in the prehospital phase, and immediate actions to stop the bleeding can save lives.^10^, ^11^, ^12^

So far, a variety of hemostats, including different types of topical dressings (e.g., sponges, sealants, and adhesives), intracavity‐injectable and expandable materials (e.g., resuscitative endovascular balloon occlusion of the aorta, Xstat, and ResQFoam), and tourniquets,^13^, ^14^, ^15^, ^16^, ^17^ have been developed for hemorrhage control in prehospital settings, but they work primarily for localized, accessible, or potentially compressible sources. For bleeding from unknown, inaccessible, or noncompressible sources, immediate interventions in prehospital settings are highly lacking. Continuous efforts have been made to address this challenge. Some recently developed hemostats, such as liquid‐infused microstructured bioadhesives,^18^ hemostatic adhesive,^19^ and injectable cryogels with rapid shape recovery,^20^ have shown promising hemostatic efficacy in preclinical bleeding models. In clinical settings, besides a few hemostatic drugs,^21^, ^22^ systemic administration of blood products (e.g. platelets, red blood cells, and fresh frozen plasma) and coagulation factors remain the primary treatment for patients with major blood loss. Though effective, blood products have many limitations for on‐site use, including donor‐dependent availability, the necessity of rigorous type‐matching, high risk of biological contamination, immunogenicity issues, short shelf‐life (e.g. 5–7 days for platelets), and stringent storage requirement.^23^, ^24^, ^25^, ^26^, ^27^, ^28^ For patients with bleeding disorders, systemic administration of defective components of the hemostatic system can generally halt bleeding and decrease mortalities.^21^ Such treatments, however, are also challenging to implement, given the difficulties in identifying the abnormalities of bleeding at the point‐of‐injury. Developing synthetic, systemic hemostatic agents for immediate intervention of bleeding from internal, non‐compressible sources, or with unknown causes is a critically unmet need.

In recent years, continued efforts have been made in the development of such synthetic, systemic hemostats, mainly through mimicking the hemostasis function of platelets via bioengineering approaches. Strategies include using substrates (i.e., particles or polymers) with ligands that can target specific molecules in the hemostasis process, such as the exposed subendothelial matrix components,^29^, ^30^, ^31^ the active integrin GPIIb‐IIIa on activated platelets,^32^, ^33^, ^34^ and the newly formed fibrin clot network.^35^, ^36^, ^37^ For instance, Arg‐Gly‐Asp (RGD) peptide has been decorated albumin microspheres^32^ and poly(lactic‐co‐glycolic acid)‐poly‐l‐lysine based nanoparticles,^33^ showing binding capability toward activated platelets and efficient hemostasis ability. Similarly, subendothelial proteins, such as collagen and von Willebrand factor (vWF), exposed to the blood at injury sites are other promising targets for this purpose. Liposomes decorated with vWF‐binding peptide (VBP), collagen‐binding peptide (CBP), and RGD‐based fibrinogen mimicking peptide have shown hemostasis efficiency in vivo.^29^, ^31^ Ligands targeting the fibrin network have also been used to develop poly(N‐isopropylacrylamide‐co‐acrylic acid) microgels‐based^36^ or polySTAT^35^, ^37^ systemic hemostats.

**FIGURE 1:**
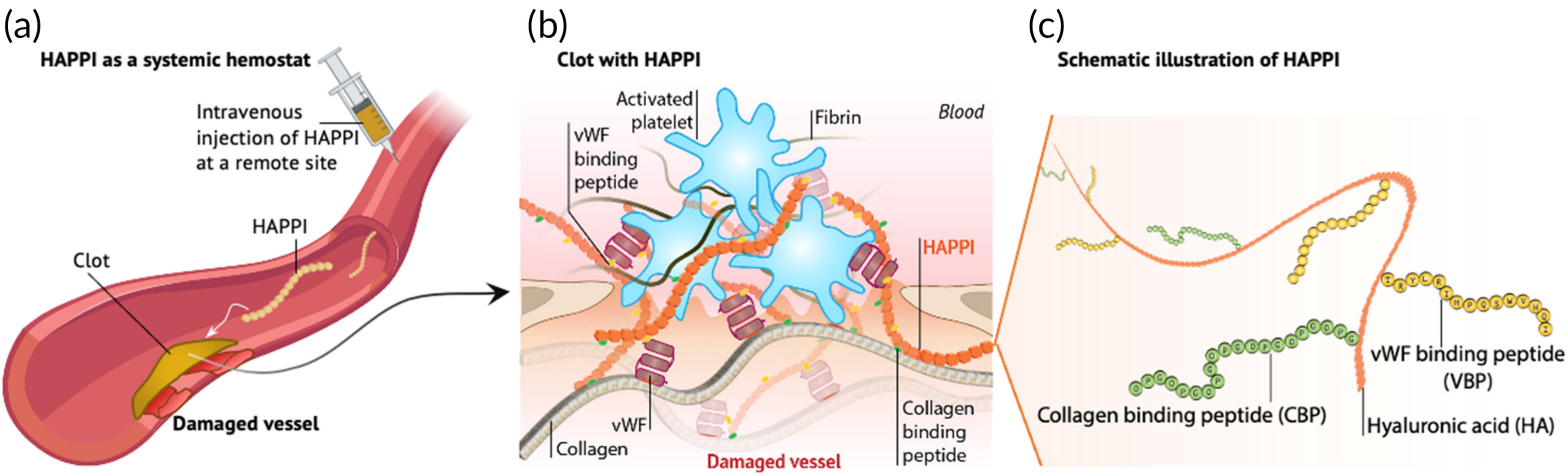
The design of HAPPI and its proposed hemostatic mechanism. After intravenous injection, HAPPI accumulates at the bleeding site (a), and promotes clot formation through its adhesion to exposed subendothelial proteins (e.g., vWF and collagen) and activated platelets (b). This is achieved via the multivalent presentation of collagen‐binding peptide and vWF‐binding peptide on hyaluronic acid (c). HAPPI, hemostatic agent via polymer peptide interfusion.

Previously, we reported a polymer‐based, systemic hemostatic agent, coined as hemostatic agent via polymer peptide interfusion (HAPPI).^38^ HAPPI was synthesized by conjugating VBP and CBP onto hyaluronic acid (HA). Bearing these two targeting peptides, HAPPI, after intravenous administration, can selectively bind to activated platelets and injury sites, promote the formation of a vascular plug, and halt bleeding within short time periods (Figure 1). In vivo studies in healthy mouse tail‐vein bleeding models showed that HAPPI can reduce blood loss by 97% compared to untreated groups without causing systemic toxicity. Here we report hemostatic efficacy of HAPPI as a field‐usable hemostat in a broad range of hemorrhagic conditions, including liver traumatic injury and uncontrollable bleeding in hemophilia, and compare these results with current clinical comparators, TachoSil® Fibrin Sealant Patch and recombinant activated factor VII (rFVIIa; NovoSeven®). To facilitate future clinical translation, we further evaluate the long‐term in vivo safety of repeatedly dosed HAPPI in mice through hematology, serum chemistry, and histological analysis.

## 2 RESULTS

### 2.1 Design of HAPPI

**FIGURE 2:**
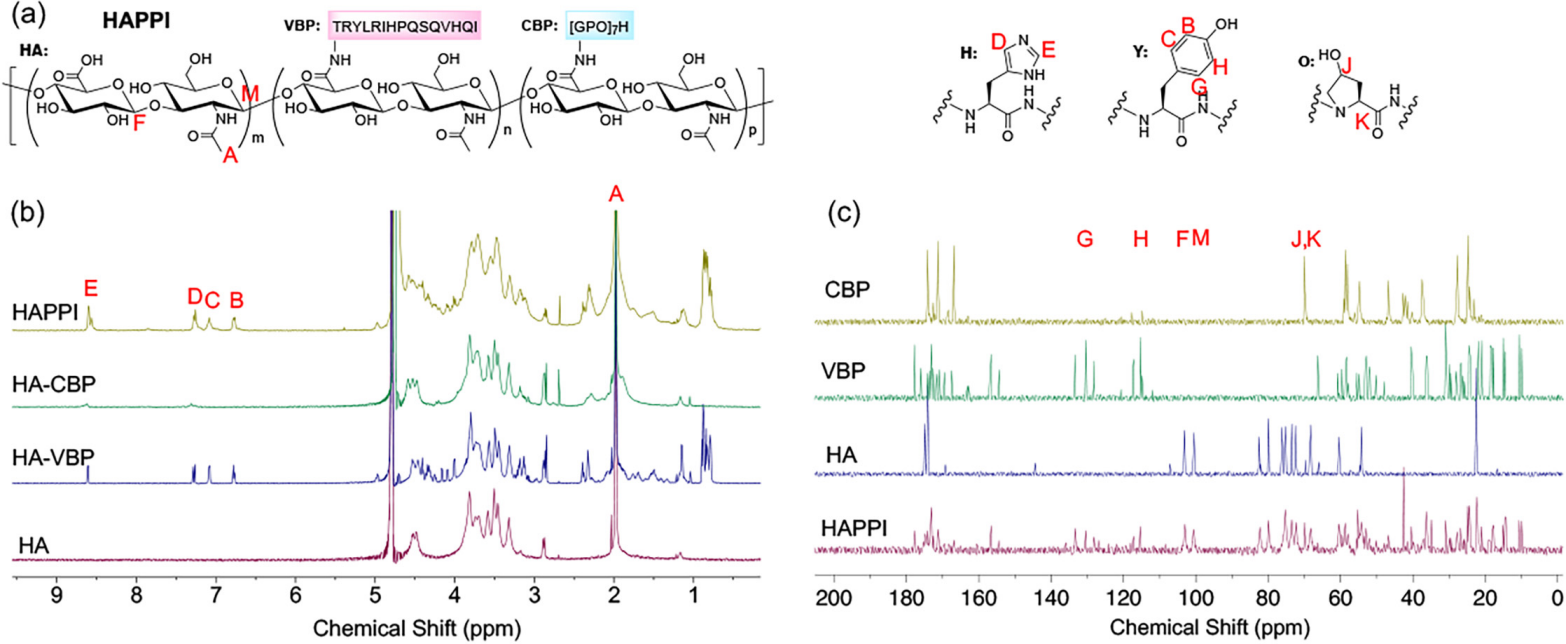
Nuclear magnetic resonance (NMR) characterization of HAPPI. (a) Chemical structure of HAPPI. (b) ^1^H NMR characteristic peaks of HA (A), VBP (B, C from tyrosine and D, E from histidine), and CBP (D, E from histidine). (c) ^13^C NMR characteristic peaks of HA (M, F), VBP (G, H from tyrosine), and CBP (J, K from hydroxyproline). HA, hyaluronic acid; HAPPI, hemostatic agent via polymer peptide interfusion; NMR, nuclear magnetic resonance; VBP, vWF‐binding peptide, CBP, collagen‐binding peptide.

Given that vWF is overexpressed at the vascular injury sites as well as on activated platelets, and that fibrillar collagen is exposed on vascular injury sites, we chose these two molecules as our targeted moieties, i.e., markers of the injury. A CBP and a VBP were chosen to covalently attach to HA, leading to the formation of HAPPI. Following our original study, we used 1‐ethyl‐3‐(3‐dimethylaminopropyl)carbodiimide (EDC)/N‐hydroxysulfosuccinimide (sulfo‐NHS)‐mediated coupling reaction to conjugate CBP and VBP to HA. The successful conjugation of VBP and CBP was validated by nuclear magnetic resonance (NMR) spectroscopy (Figure 2) and Fourier‐transform infrared spectroscopy (FTIR, Figure S1). The FTIR spectrum of HAPPI includes the characteristic bands from HA, such as 3322, 2880, and 1041 cm^−1^ corresponding to O—H, C—H and C—O stretching vibrations, respectively. The C—H bending vibrations of HA in the range of 1404–1300 cm^−1^ in the HAPPI are also shown in the HAPPI spectrum. Characteristic peaks from peptides are also visible in the HAPPI spectrum but partly overlap with HA. Compared to HA, the most noticeable change is the presence of peptide bonds (e.g., 1634 and 1553 cm^−1^). The stretching vibrations of C—O in carboxylate anion from HA (in 1603 cm^−1^) are not visible after conjugation, suggesting that the carboxylic group was the reaction site, but the newly formed amide bond is overlapped with those from peptides.

This design of HAPPI with the multivalent presentation of VBPs and CBPs, targets them to the vascular injury sites, recruits activated platelets, and promotes their adhesion, thereby promoting the formation of a vascular plug and inducing hemostasis (Figure 1). The highly localized presence of the targeted molecules (e.g., vWF and collagen) ensures that these events occur only at the injury sites without causing systemic clotting. We posit that this facilitates HAPPI as a safe and effective treatment strategy with high translational feasibility for life‐threatening, noncompressible traumatic bleeding.

### 2.2 Systemic treatment of traumatic hemorrhage in the rat liver laceration model

**FIGURE 3:**
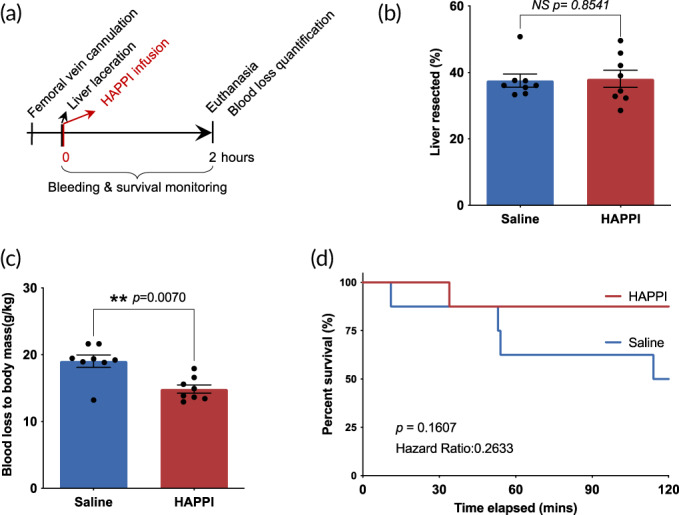
Hemostatic effect of HAPPI in the systemic treatment of traumatic bleeding in rat liver transection model. (a) In vivo experimental outline: the femoral vein was cannulated, and HAPPI solution or saline was infused immediately after the induction of bleeding through liver laceration. Rats were monitored for 2 h and euthanized after that. (b) Percent of median liver resected was quantified to compare the injury severities. Hemostatic efficacy of HAPPI was evaluated by quantifying total blood loss (c) and percentage of survival (d). Saline and HAPPI solution were infused at volume dose of 1 mL/kg. Dosages of HAPPI were 12 mg/kg. All data are means ± SEM. Statistical significance was determined using unpaired, two‐tailed Mann–Whitney test (NS: no significance; **p < 0.01) (b and c) and Gehan–Breslow–Wilcoxon test (d). HAPPI, hemostatic agent via polymer peptide interfusion.

HAPPI, dissolved in a saline solution, was intravenously administrated to rats injured by a hepatic resection procedure (Figure 3). To mimic the use as a rescue treatment at the site of injury, HAPPI was infused after the resection (Figure 3a). Compared to the saline‐treated group, the treatment of HAPPI promoted the clot formation with a significant reduction in both blood loss and mortality rate (Figures 3c,d, Table 1). Specifically, intravenous infusion of HAPPI immediately after the initiation of hemorrhage, significantly decreased the normalized blood loss from 19.0 g/kg (in saline group) to 14.9 g/kg (*p* = 0.0070) for similar liver resection degrees (~38%, Figure 3b). Additionally, seven out of eight rats treated with HAPPI survived for the 2‐h monitoring period, while only four out of eight rats survived in the saline treated group. Taken together, the decrease in blood loss and the increase in survival rates indicate that faster hemostasis was achieved in this lethal trauma model in rats treated with HAPPI compared to saline‐treated ones.

**TABLE 1:** Univariate statistical analysis comparing the saline group with the HAPPI group using the Mann–Whitney test.

Variable	Saline group	HAPPI group	*p*‐values
Number of animals	8	8	
Body weight (g)	353.0 (316.8–416.3)	360.4 (322.3–409.8)	0.5737
Total liver weight (g)	12.6 (10.7–13.5)	13.1 (12.0–16.4)	0.8165
Liver resected (g)	4.7 (3.6–6.6)	5.0 (3.2–6.4)	0.8573
Liver resected (%)	37.6 (33–50)	38.1 (28–49)	0.8541
Blood loss (g)	6.8 (4.2–8)	5.4 (4.2–6.8)	0.0466
Blood loss (g/kg)	19.0 (13–22)	14.9 (13–18)	0.0070
Median blood loss/median liver weight	0.53 (0.39–0.59)	0.41 (0.34–0.48)	n/a
Mortality	4 (50%)	1 (12.5%)	n/a
Survival time (min)	89 (11–120)	109 (34–120)	0.1607^a^

^a^ *p* value was obtained using Gehan–Breslow–Wilcoxon test of Kaplan–Meier curves.

### 2.3 Hemostatic efficacy of locally applied HAPPI in the rat liver punch biopsy model

**FIGURE 4:**
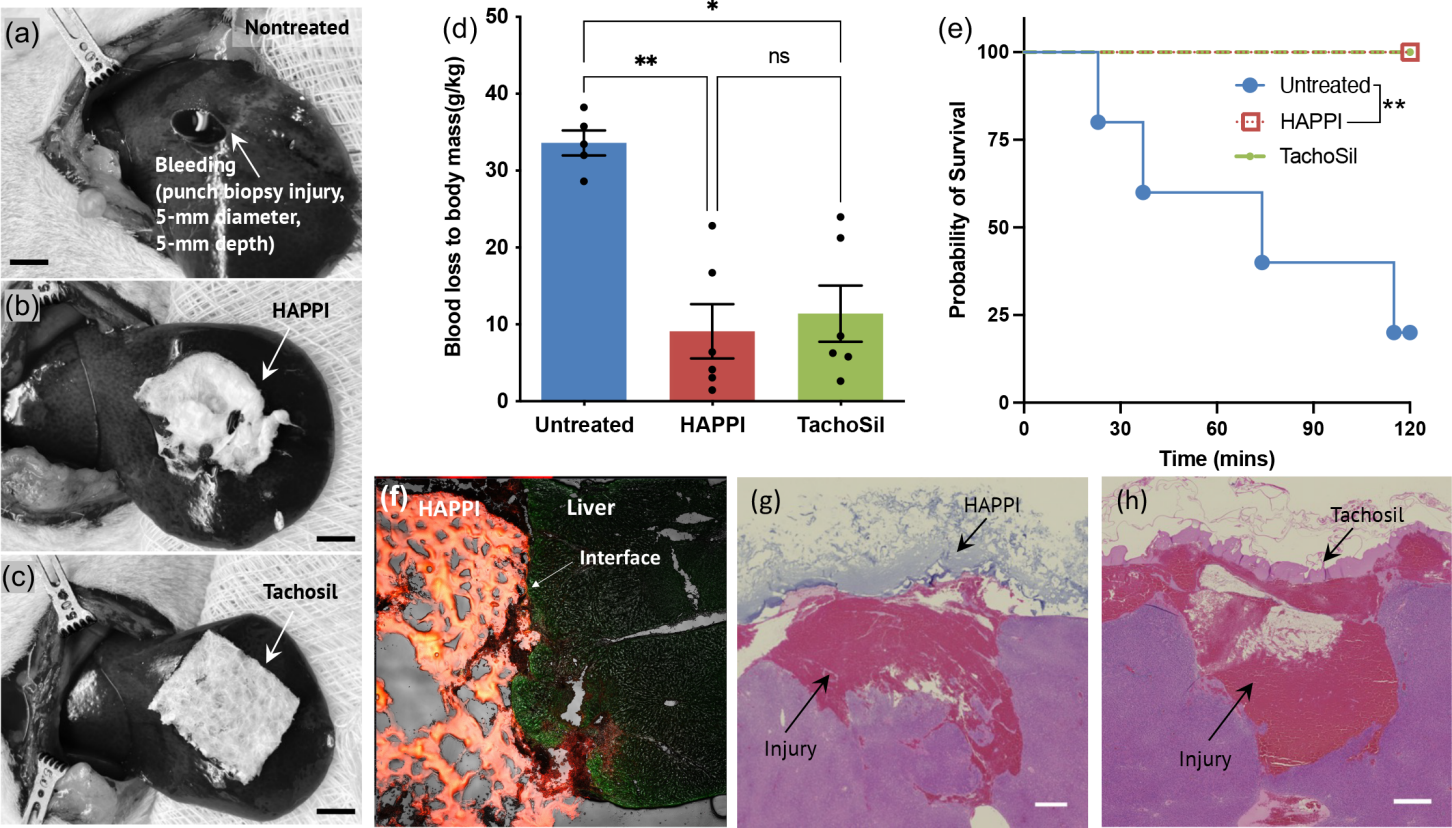
Hemostatic efficacy of topical applied HAPPI forms in rat liver punch biopsy model. (a) Untreated liver injury induced via punch biopsy. Topical application of HAPPI forms (b) and TachoSil patch (c) to the bleeding site. Rats were heparinized (250 IU/kg) prior to injury. Scale bar: 5 mm. (d and e) Hemostatic efficacy was evaluated by quantifying total blood loss (d) and survival (e). (f) Representative fluorescence images for injured liver with topical applied HAPPI forms (red). (g and h) Representative histology images stained with hematoxylin and eosin (H&E) for injured liver with topical applied HAPPI (g) and TachoSil (h). Scale bar: 100 μm. All data are means ± SEM. Statistical significance was determined using Kruskal–Wallis with Dunn's multiple comparisons test (*p < 0.05, **p < 0.01, ns: no significance) (d) and Gehan–Breslow–Wilcoxon test (**p < 0.01) (e). HAPPI, hemostatic agent via polymer peptide interfusion.

We further evaluated the hemostatic efficacy of HAPPI for local application. Since HAPPI can be stored and supplied as a dry solid material, for traumatic bleeding from external and accessible sources, direct application of HAPPI solid is a feasible option. For this purpose, we used a liver punch biopsy model in heparinized rats with TachoSil® Fibrin Sealant Patch as the commercial comparator. Topical application of lyophilized HAPPI solids onto the bleeding site resulted in a significant reduction in blood loss and an increase in survival rate (Figure 4a–e). Compared to the untreated group, a 73% decrease in median blood loss was observed in the HAPPI group, comparable to the TachoSil‐treated group. Moreover, all animals treated with HAPPI survived during the 2‐h monitoring period. In contrast, only one out of five animals survived in the untreated group. Upon topical application, HAPPI likely underwent in‐situ dissolution after contacting blood or other biofluids at the bleeding site. Solvated HAPPI at the application site likely promoted hemostasis as in the case of systemic administration. As shown in Figure 4f, firm adhesion between HAPPI and liver tissue was achieved, and the penetration of solvated HAPPI, labeled by Alexa‐Flour 647, into the liver injury was observed. Histopathological analysis of hematoxylin and eosin (H&E)‐stained tissue sections did not show inflammation or toxicity of HAPPI to the liver tissue (Figure 4g) or other vital organs (Figure S2). The retention of blood clots inside the injury cavity further confirmed the firm attachment of HAPPI to the injured tissues, as also seen in the TachoSil‐treated group (Figure 4h).

### 2.4 Hemostatic efficacy in tail‐vein bleeding in hemophilia A mice

**FIGURE 5:**
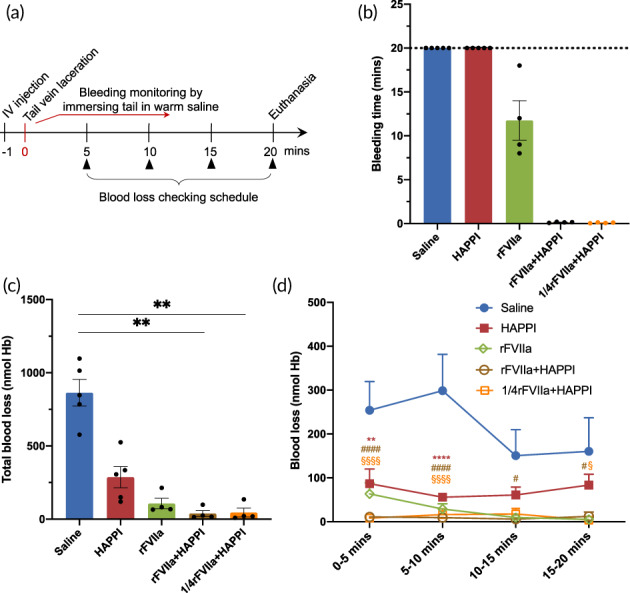
Hemostasis efficacy of HAPPI in the absence or presence of recombinant factor VIIa in a tail‐vein bleeding model in hemophilia A mice. (a) In vivo experimental outline: mice were injected with control or experimental formulations. One minute later, teil‐vein bleeding was induced using the tail‐vein laceration model. Mice were monitored for 20 min and euthanized at 20 min. Hemostatic efficacy of HAPPI was evaluated by quantifying bleeding time (b), total blood loss (c), and blood loss at different time intervals (d). Saline and experimental formulations were injected at a volume dose of 5 mL/kg. Dosages of HAPPI in HAPPI, rFVIIa/HAPPI, and 1/4rFVIIa/HAPPI groups were fixed at 24 mg/kg. Dosage of rFVIIa in rFVIIa, rFVIIa/HAPPI, and 1/4rFVIIa/HAPPI groups were 2.7, 2.7, and 0.675 mg/kg, respectively. All data are means ± SEM. (c) Kruskal–Wallis with Dunn's multiple comparisons test (**p < 0.01). (d) Mixed‐effects model (matched values are both stacked and spread across a row) and Dunnett's multiple comparison test with a single pooled variance (****, ####, §§§§*p* < 0.0001, **p < 0.01, *, #, §*p* < 0.05). HAPPI, hemostatic agent via polymer peptide interfusion.

HAPPI was next evaluated for hemostatic efficacy in hemophilia A mice. Bleeding from hemophilia, a bleeding disorder caused by deficiency of coagulation factor VIII (hemophilia A) or factor IX (hemophilia B), could be life‐threatening without prompt interventions.^7^ While hemophilia treatment options had expanded significantly including various prophylactic and on‐demand treatment agents, for hemophilia patients with inhibitors, breakthrough bleeding episodes treatment is still limited to bypassing agents such as recombinant activated human FVII.^39^ Synthetic materials that can help stop bleeding can add significantly to the arsenal to treat hemophilia patients. For this study, we used a previously reported tail‐vein laceration bleeding model in hemophilia A mice.^40^ Mice treated with saline, as a negative control, failed to achieve hemostasis during the 20‐min observation period (Figure 5a,b), which is inconsistent with other published reports in tail‐vein bleeding models in hemophilia mice.^40^, ^41^ While the administration of HAPPI at a dose of 24 mg/kg did not halt bleeding within 20 min, the bleeding was much slower compared to the saline group, with a significant reduction (approx. three times) in averaged blood loss (Figure 5c). This is expected, since HAPPI presumably only involved in the primary hemostasis process through its adhesion to activated platelets, vWF, and collagen to form the platelet plug,^38^ while hemophilia treatment needs to restore the secondary hemostasis as demonstrated in the rFVIIa‐treated group (at a dose of 2.7 mg/kg). Remarkably, the administration of combined HAPPI (24 mg/kg) and rFVIIa (2.7 mg/kg) led to immediate hemostasis. Within the 20‐min monitoring period, the rFVIIa+HAPPI formulation resulted in a 95% reduction in total blood loss compared to the saline‐treated group (Figure 5c). Compared to the rFVIIa‐treated group, the standard of care for on‐demand treatment of hemophilia, the combination of HAPPI with rFVIIa reduced the blood loss by 83% within the first 5‐min phase and 64% for the entire monitoring period (Figure 5c,d).

With the aim to reduce the cost of the final formulation, we further reduced the dose of rFVIIa by four times with the dose of HAPPI unchanged. As shown in Figure 5b,c, immediate hemostasis and a reduced blood loss were observed. In the lower dose rFVIIa combinational formulation, two out of four mice displayed very slow rebleeding episodes at 5 min and 17 min, respectively. Nonetheless, these data demonstrated beneficial of combining HAPPI with rFVIIa in treating bleeding in hemophiliac subjects.

### 2.5 Biocompatibility and long‐term safety

**FIGURE 6:**
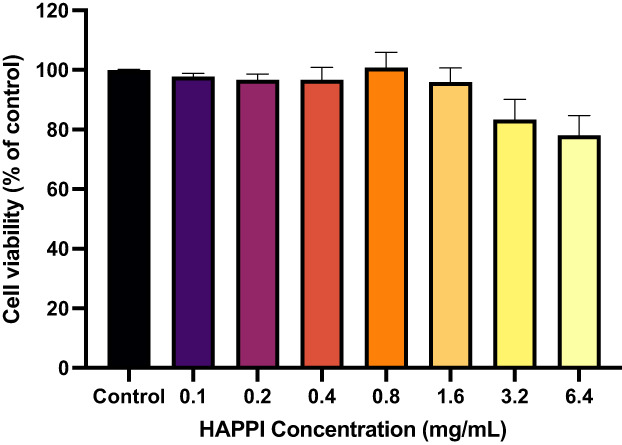
In vitro cell viability study of HAPPI. MTS assay of HUVEC cells incubated for 24 h with HAPPI at 0.1–6.4 mg/mL. Data are shown as the percentage of viable cells compared to the control group (100%) and values are given as mean ± SEM (*n* = 4). No statistical difference was found between HAPPI‐treated groups and the control group. Statistical analysis was performed by Kruskal–Wallis with Dunn's multiple comparisons test (ns, *p* > 0.05). HAPPI, hemostatic agent via polymer peptide interfusion; HUVEC, human umbilical vein endothelial cell.

**FIGURE 7:**
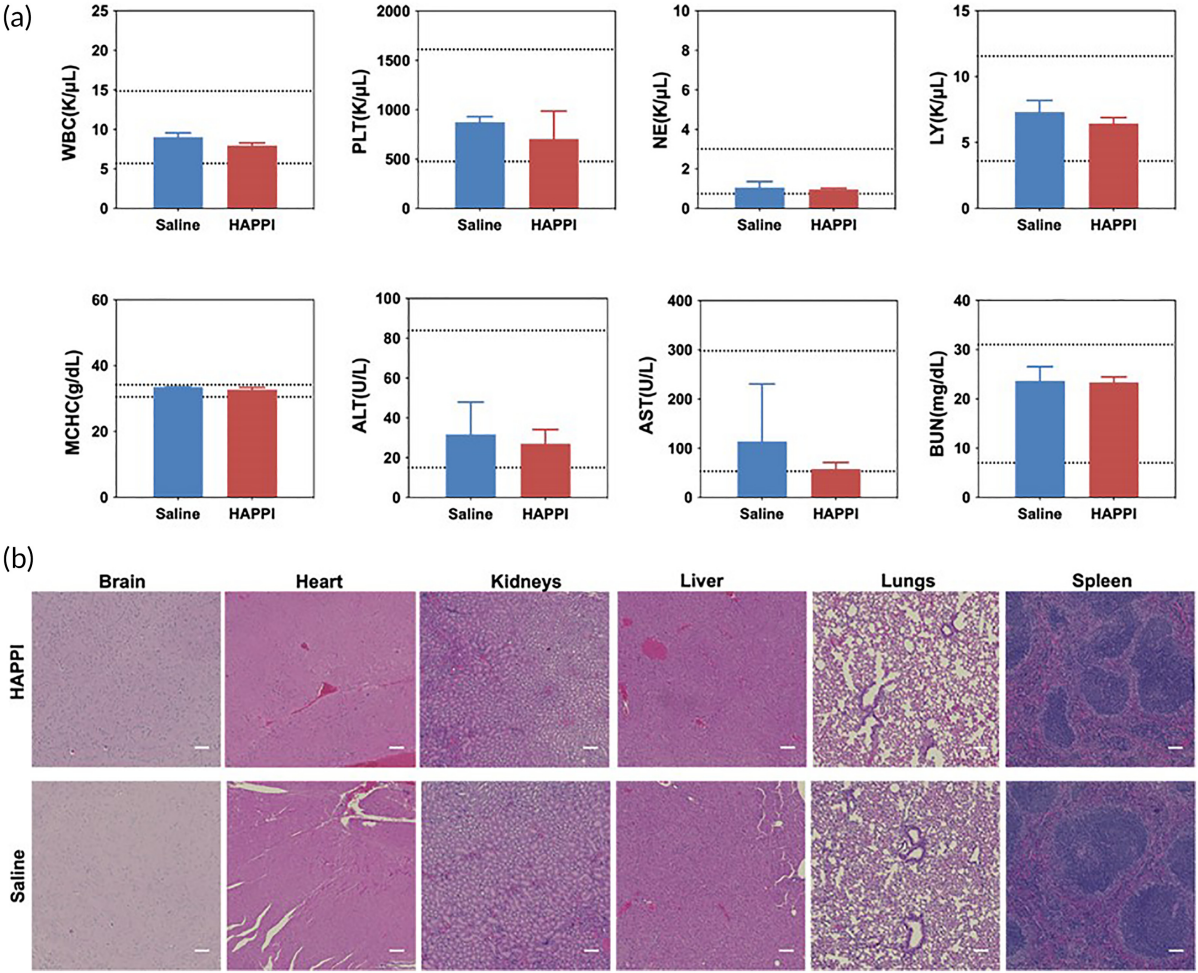
In vivo safety study of HAPPI. HAPPI and saline (in the control group) were repeatedly dosed on Days 0, 30, 60, and 90 and blood and organs were collected on Day 97 for hematology and serum chemistry analysis and histological analysis, respectively. (a) Hematology and serum chemistry analysis. Abbreviations: WBC: white blood cells; PLT: platelets; NE: neutrophils; LY: lymphocytes; MCHC: mean corpuscular hemoglobin concentration; ALT: alanine aminotransferase; AST: aspartate aminotransferase; BUN: blood urea nitrogen. All data are mean ± SD. *n* = 3. Dashed lines indicate established normal ranges (two lines).^42^, ^43^, ^44^ (b) Representative images of hematoxylin and eosin (H&E) staining of vital organs in HAPPI‐ and saline‐treated mice (scale bar: 100 μm). HAPPI, hemostatic agent via polymer peptide interfusion.

The cytocompatibility of HAPPI was evaluated in vitro using 3‐(4,5‐dimethylthiazol‐2‐yl)‐5(3‐carboxymethonyphenol)‐2‐(4‐sulfophenyl)‐2H‐tetrazolium (MTS) assay in human umbilical vein endothelial cells (HUVECs). HAPPI with concentrations ranging from 0.1 to 6.4 mg/mL was incubated with HUVECs for 24 h. No statistical difference in cell viability was observed between HAPPI treated groups and the untreated control group, although a modest decrease in percentage of viable cells was observed for HAPPI with concentration over 3.2 mg/mL (Figure 6). To further evaluate the in vivo safety of HAPPI, we performed long‐term, repeated intravenous injections of HAPPI (on Days 1, 30, 60, and 90) into healthy mice at the dose that was previously validated to be effective for hemostasis.^38^ Blood collected on Day 97 was subjected to hematological and biochemistry analyses. Figures 7a and S3 showed that all measured parameters in blood compositions were similar between HAPPI and saline groups and were in the normal range. In particular, HAPPI treatment did not change platelet and white blood cell (including lymphocyte and neutrophil subsets) counts and mean corpuscular hemoglobin concentration. The liver transaminase (i.e., alanine aminotransferase and aspartate aminotransferase) levels and the blood urea nitrogen (a marker of kidney function) in serum were unaffected in the HAPPI‐treated group. These results indicate that the repeated, long‐term treatment of HAPPI did not cause detectable systemic toxicity. We further confirmed the safety of HAPPI through histopathological analysis of hematoxylin and eosin (H&E)‐stained sections of vital organs harvested on Day 97. Same to the saline group, HAPPI treatment did not cause inflammation or toxicity in any of the vital organs (Figure 7b). Overall, this confirmed that HAPPI is safe and blood compatible in mice after repeated and long‐term administration, which is consistent with our previous study.^38^

## 3 DISCUSSION

Systemic hemostatic agents based on synthetic materials hold great promise in addressing the current challenge in the immediate management of internal, noncompressible bleeding,^16^ or bleeding with unknown sources.^45^ The impact of such agents could be even greater when their underlying hemostatic mechanism works for a broad range of hemorrhagic conditions (e.g., traumatic bleeding and hemophilia). HAPPI is one such synthetic, systemic hemostat that mainly targets to the primary hemostasis step.^38^ As the first and essential step toward clot formation, primary hemostasis includes the adhesion (to collagen and vWF exposed at injury sites), activation, and aggregation of platelets, leading to the formation of platelet plugs.^15^ The two adhesion peptides, CBP and VBP, on HAPPI allow it to selectively bind to vWF and collagen at vascular injury sites. By further attracting activated platelet, the accumulated HAPPI can augment the formation of vascular plugs, and thus induce hemostasis (Figure 1). The highly localized presence of the targeted molecules (i.e., vWF and collagen) ensures that these events occur only at the injury sites without causing systemic clotting.

This mechanism of action makes HAPPI a promising agent for treating different hemorrhagic conditions, particularly for those benefiting from enhanced and accelerated primary hemostasis. For hemorrhage from minor injuries (e.g., mice tail‐vein bleeding model), intravenously administered HAPPI can staunch bleeding instantaneously without causing systemic thrombosis and toxicity.^38^ Here, we further explored the potential of HAPPI as field‐usable hemostats in treating severe, lethal bleeding episodes, such as for trauma patients and hemophiliacs. In a rat liver laceration model, intravenous injection of HAPPI results in a significant decrease in blood loss from 19.0 g/kg (in the saline group) to 14.9 g/kg (*p* = 0.0070) with a mortality rate decrease from 50% to 12.5% within the 2‐h monitoring period after injury. This nonsurgical hemostasis strategy for fatal hepatic hemorrhage is of clinical significance, given that liver is one of the most frequently injured organs in abdominal trauma,^46^, ^47^ and uncontrolled hemorrhage is the key cause of liver injury‐related casualty with a mortality rate up to 80%.^48^, ^49^ In case of bleeding episodes from hemophilia A mice, compared to the saline group, HAPPI‐treated mice showed much slower bleeding and three‐fold less blood loss with the 20‐min monitoring period, presumably due to the enhanced primary hemostasis step. This was further improved by also restoring the secondary hemostasis by introducing rFVIIa into the HAPPI formulation. The combination of rFVIIa and HAPPI led to rapid hemostasis and a 95% reduction in total blood loss compared to the saline‐treated group. The combination therapy also reduced the blood loss by 83% within the first 5‐min phase and 64% for the entire monitoring period, compared to the rFVIIa group.

HAPPI has hemostatic potential for traumatic bleeding from external as well as accessible sources. The rationale here is that the primary action of HAPPI is to rapidly interact with activated platelets and subendothelial proteins at injury sites. Also, HAPPI can be stored and supplied as a dry solid, and only to be reconstituted at the site of injuries. Direct application of HAPPI solids for external or accessible wounds would be another efficient way for hemorrhage control. In a liver punch biopsy model in heparinized rats, HAPPI solids were applied locally at the injury site, yielding a 73% of reduction in blood loss and a significant increase in survival rate (from 20% in the untreated group to 100%). This could be attributed to the in‐situ dissolution of HAPPI at the wound site, concentrating the coagulation components, and the solvated HAPPI promotes hemostasis as in the case of systemic administration. It should be mentioned that HA used in synthesizing HAPPI is critical for the subsequent wound healing process,^15^, ^17^ further highlighting HAPPI's promise as an emerging multi‐faceted hemostat. However, certain technological challenges must be overcome to translate HAPPI into the clinic. One challenge, as is the case for other polymer‐based therapeutics,^50^ is the structural heterogeneity, originating from the polydisperse nature of HA, uncontrolled peptide conjugation sites, and varied conjugate degree. Use of monodispersed polymers with defined functionality offers a potential solution to overcoming this structural heterogeneity. Species variation in protein structures (e.g., vWF and collagen) and coagulation functions represents another challenge for the preclinical‐to‐clinical transition of HAPPI. So far, CBP and VBP used in the construction of HAPPI are validated to be effective for hemostasis in animal bleeding models, but their performance in treating hemorrhage in human patients remains to be verified.

## 4 CONCLUSION

Our results demonstrate that HAPPI is highly effective in treating lethal traumatic and hemophilic bleeding via either systemic administration or topical application. Intravenous injection of HAPPI resulted in a significant decrease in blood loss in both the rat liver traumatic model and tail‐vein laceration model in hemophilia A mice. Topical application of HAPPI on liver punch biopsy wounds in heparinized rats achieved a significant reduction in blood loss and an increase in survival rate, compared to the saline‐treated group. HAPPI represents a promising hemostatic agent for a broad range of different hemorrhagic conditions.

## 5 MATERIALS AND METHODS

### 5.1 Synthesis and characterization of HAPPI

The synthesis and characterization of HAPPI followed our previous study.^38^ Briefly, HA (250 kDa, Creative PEGWorks, Chapel Hill, NC) was dissolved in a 1:1 mixture of DI water and DMSO at 7.5 mg/mL. Sulfo‐NHS (Sigma‐Aldrich, St. Louis, MO, 2× molar excess of peptides) was dissolved in DI water at 150 mg/mL, and EDC·HCl (Sigma‐Aldrich, St. Louis, MO, 2× molar excess of peptides) was dissolved in DMSO at 50 mg/mL. Sulfo‐NHS and EDC·HCl solutions were added to the HA solution and stirred for 1 h at room temperature. CBP ([GPO]_7_H, GenScript USA Inc., Piscataway, NJ) and VBP (TRYLRIHPQSQVHQI, GenScript USA Inc., Piscataway, NJ) solutions (in DMSO at 50 mg/mL, 10 mol %) were then added to the reaction solution for overnight reaction at room temperature. The conjugate products were purified by dialysis against a large excess amount of DMSO/DI water solution for 3 days (50, 30, and 10% DMSO solution, each for 1 day) and pure DI water for another 4 days (solvent renewal: twice daily). The conjugates were then freeze‐dried and stored at −20°C until further use. For NMR characterization, the conjugates were dissolved in deuterated water, and ^1^H and ^13^C NMR spectra were obtained on Agilent DD2 600 MHz NMR Spectrometer (Santa Clara, CA) and Bruker 400‐MHz NMR Spectrometer (Fremont, CA), respectively with MNova 10.0.1 (Mestrelab Research, Spain) processing software. FT‐IR spectra were obtained using a Nicolet iS‐50 FT‐IR Spectrometer and analyzed using OPUS software.

### 5.2 Biocompatibility study

EA.hy926 cells (ATCC) were grown in DMEM supplemented with 10% FBS. Cultures were maintained in a humidified atmosphere at 5% CO_2_ at 37°C. The cells were seeded in a 96‐well plate at a seeding density of 10,000 cells/well. Twenty‐four hours after seeding, media was removed, and the cells were treated with media containing HAPPI (concentrations ranging from 0.1 mg/mL to 6.4 mg/mL) versus media without HAPPI (*n* = 4 for all test groups). The treated cells were incubated at normal culture conditions for 24 h. After 24 h, a standard MTS assay (CellTiter 96 Aqueous One Solution Cell Proliferation Assay, Promega) was used to assess the metabolic viability of the cells. This assay is a colorimetric method for determining the number of viable cells in proliferation. Following 24 h of incubation with or without HAPPI, the treatments were removed, and MTS solution was applied to all wells. The cells were incubated at normal culture conditions for 2 h. After 2 h, absorbance was recorded at 490 nm using plate reader.

### 5.3 In vivo hemostasis and safety studies

All animal experiments were performed as per protocols approved by the Institutional Animal Care and Use Committee of Harvard University (IACUC_17–04–299‐1).

#### 5.3.1 Rat liver laceration model

Sixteen Sprague–Dawley rats (male, 8–10 weeks old, Charles River Laboratories) were randomized into two groups (eight rats each): saline and HAPPI. HAPPI was formulated by dissolving it in sterile saline (Teknova, Hollister, CA), and dosed at 12 mg/kg. Rats were anesthetized with isoflurane (2%–3%) and kept under isoflurane anesthesia for the entire surgery and monitoring period. Following the incision at the left inguinal, the left femoral vein was isolated from the connected tissue using a Size 3 suture and cannulated with a 22‐gauge catheter with prefilled saline. The catheter was flushed with 50 μL of saline to avoid blood coagulation inside the catheter. Subsequently, the abdomen was entered via a midline incision. The liver was exposed, and the left and right lateral lobes were resected at around 2.5 cm from the edge. Immediately following the resection, saline (1 mL/kg) or HAPPI (1 mL/kg) was infused through the femoral vein catheter. This was the time zero, and the rat was monitored for another 2 h or until death, whichever came first. The mortality was determined by the cessation of respiratory movements. Anesthetic depth was checked throughout the monitoring period. Survival time was recorded. Rats that survived for the 2‐h monitoring period were euthanized through CO_2_ inhalation. The abdomen was re‐opened, and blood loss was quantified using preweighed gauze.

#### 5.3.2 Rat liver punch biopsy model

Seventeen Sprague–Dawley rats (male, 8–10 weeks old, Charles River Laboratories) were randomized into three groups: untreated, HAPPI, and TachoSil. Similar to the liver laceration model as described above, the left femoral vein was cannulated under isoflurane anesthesia. The liver was exposed after a midline incision. Right before liver injury, heparin solution (250 IU/kg) was infused via the catheter. The left lateral liver lobe was injured by a 5‐mm punch biopsy (Integra Miltex, Plainsboro, NJ) with a depth of 5 mm. The liver core was removed, and rats in the untreated group was bleeding freely. For the HAPPI and TachoSil group, lyophilized HAPPI form and TachoSil patch were trimmed to approximately 1 × 1 cm and applied to the injury site immediately following the injury. Rats were monitored for 2 h or until death, whichever came first. The survival time was recorded, and blood loss was quantified, as described in the liver laceration model section.

#### 5.3.3 Tail‐vein bleeding model in hemophilia mice

To evaluate the hemostatic efficacy of HAPPI in hemophilia models, mutant mice with severe factor VIII deficiency (less than 1% of wild‐type levels) were used (B6;129S‐*F8* ^*tm1Kaz*^/J, Female, 8–10 weeks, Jackson Laboratory, stock #004424). The tail‐vein bleeding model in these mice was established based on a previously reported procedure^38^, ^40^ with sight modification. Briefly, mice were randomized into different groups, including saline, HAPPI, rFVIIa, and rFVIIa/HAPPI. Formulations with a dose volume of 5 mL/kg were administered via IV bolus injection through tail vein using 27‐gauge needles. The dosage of experimental formulations, as detailed in the caption of Figure 5, was determined based on previous reports.^38^, ^41^ For rFVIIa/HAPPI formulations, equal volumes of rFVIIa and HAPPI solutions were mixed to yield the desirable final concentrations. As shown in Figure 5a 1 min after the injection, tail‐vein laceration was made on another lateral vein (other than the injected vein) using the tail‐vein laceration template^40^ and a #10 scalpel. Immediately after the laceration, the injured tail was placed in a 15 mL falcon tube containing 14 mL of pre‐warmed saline at 37°C. Bleeding was monitored for another 20 min, and the falcon tube with warm saline was replaced with a new one every 5 min to calculate the blood loss at different time intervals. Animals were euthanized 20 min after laceration. The blood loss was determined by quantifying the hemoglobin using the Drabkin's reagent (D5941, Sigma‐Aldrich, St. Louis, MO), as reported previously.^38^

#### 5.3.4 Long‐term toxicity studies in healthy mice

Ten healthy BALB/c mice (female, 20 g) were randomly allocated to two groups (*n* = 5): HAPPI and saline control. HAPPI (reconstituted in saline) or saline was intravenously administered on Days 1, 30, 60, and 90. On Day 97, blood was withdrawn, and mice were sacrificed. Vital organs including the brain, heart, kidneys, liver, lungs, and spleen were harvested and fixed by formalin for histological analysis. Organ sections were stained with H&E. Blood samples were submitted to IDEXX Laboratories (North Grafton, MA) for hematological and biochemistry analyses. For hematological analyses, whole blood was collected into a 0.5‐mL EDTA anticoagulant tube, mixed by gently inverting the tube to prevent clotting and hemolysis, and stored on ice. For biochemistry analyses, blood samples were collected into a 0.8‐mL serum gel tube, and serum was separated by centrifugation at 2000g for 10 min after 20 min of clotting at room temperature.

## AUTHOR CONTRIBUTIONS

**Yongsheng Gao:** Conceptualization (lead); data curation (lead); formal analysis (lead); investigation (lead); methodology (lead); writing – original draft (lead); writing – review and editing (equal). **Mayumi Ikeda‐Imafuku:** Methodology (supporting). **Zongmin Zhao:** Formal analysis (supporting); methodology (supporting). **Maithili Joshi:** Formal analysis (supporting); methodology (supporting). **Samir Mitragotri:** Conceptualization (equal); funding acquisition (lead); writing – review and editing (equal).

## CONFLICT OF INTEREST STATEMENT

S.M. and Y.G. are inventors on a patent application that covers some aspects of the technology reported in this manuscript (owned and managed by Harvard University).

## Supporting information


**Data S1.** Supporting Information.

## Data Availability

The data that supports the findings of this study are available in the supplementary material of this article.
