# Supplementary material for: A polymer‐based systemic hemostat for managing uncontrolled bleeding
Source: Bioeng Transl Med. 2023 Apr 13;8(3):e10516. doi: 10.1002/btm2.10516 (PMC10189483; doi:10.1002/btm2.10516)
Supplement: Supplementary file 1 — Data S1. Supporting Information. [file BTM2-8-e10516-s001.pdf]

A polymer-based systemic hemostat for managing uncontrolled bleeding

Yongsheng Gao,[1,2] Mayumi Ikeda-Imafuku,[1,2] Zongmin Zhao,[1,2] Maithili Joshi,[1,2] Samir Mitragotri[1,2]*

[1] John A. Paulson School of Engineering and Applied Sciences, Harvard University, Allston, MA 02134, USA.

[2] Wyss Institute for Biologically Inspired Engineering at Harvard University, Boston, MA 02115, USA.

*Corresponding author. Email: mitragotri@seas.harvard.edu

SUPPLEMENTARY MATERIALS

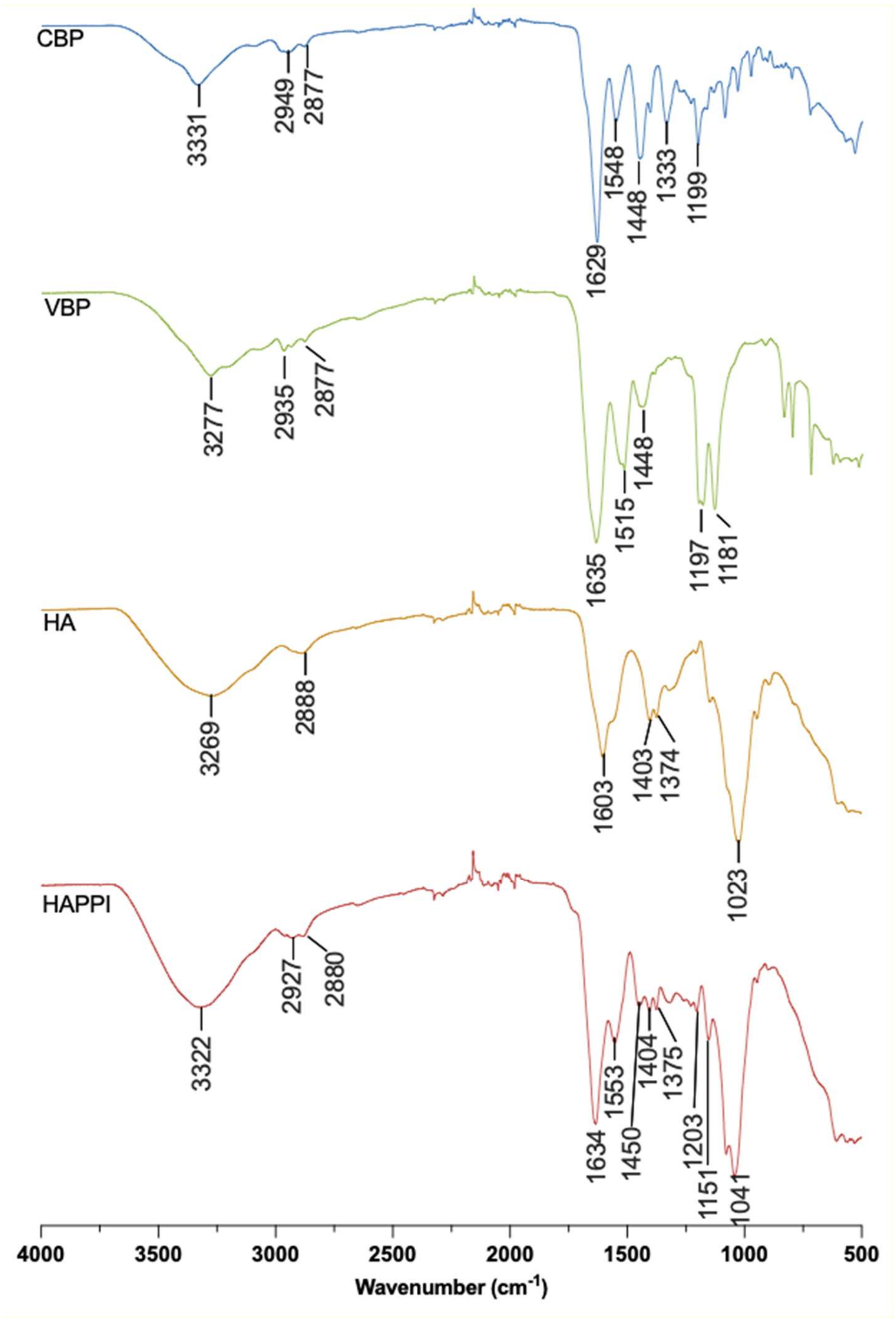

Figure S1. FTIR spectra of CBP, VBP, HA, HAPPI.

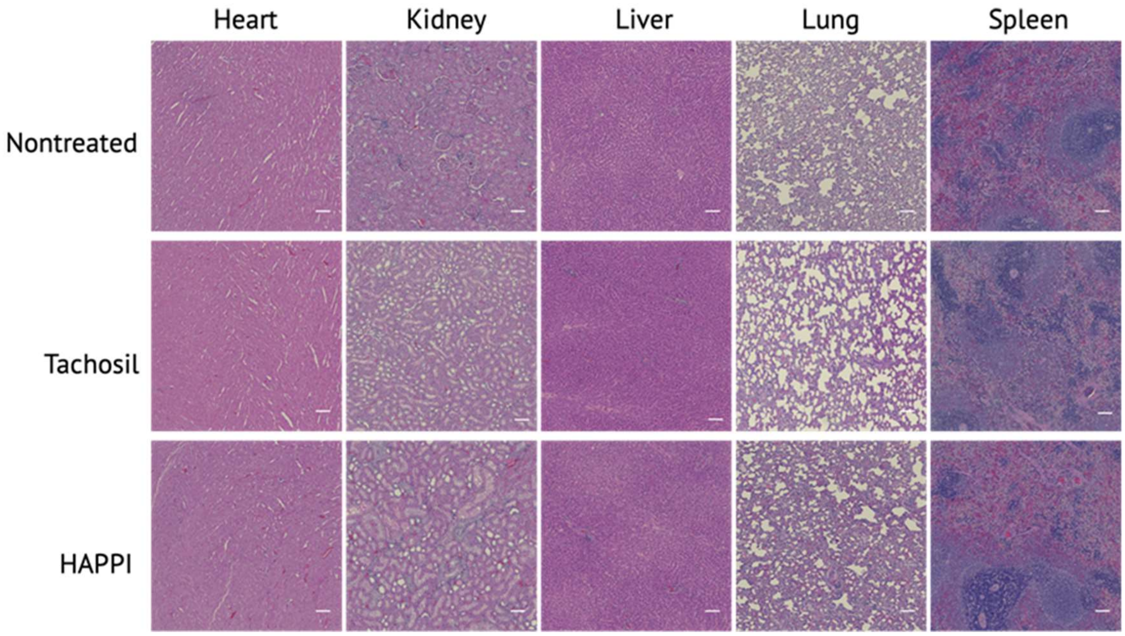

Fig. S2. Representative graphs of hematoxylin and eosin (H&E) staining of vital organs of untreated rats, TachoSil-treated and HAPPI-treated rats with liver punch biopsy injuries. Organs were harvested at 2 hours after the injury. (Scale bar: 100 µm)

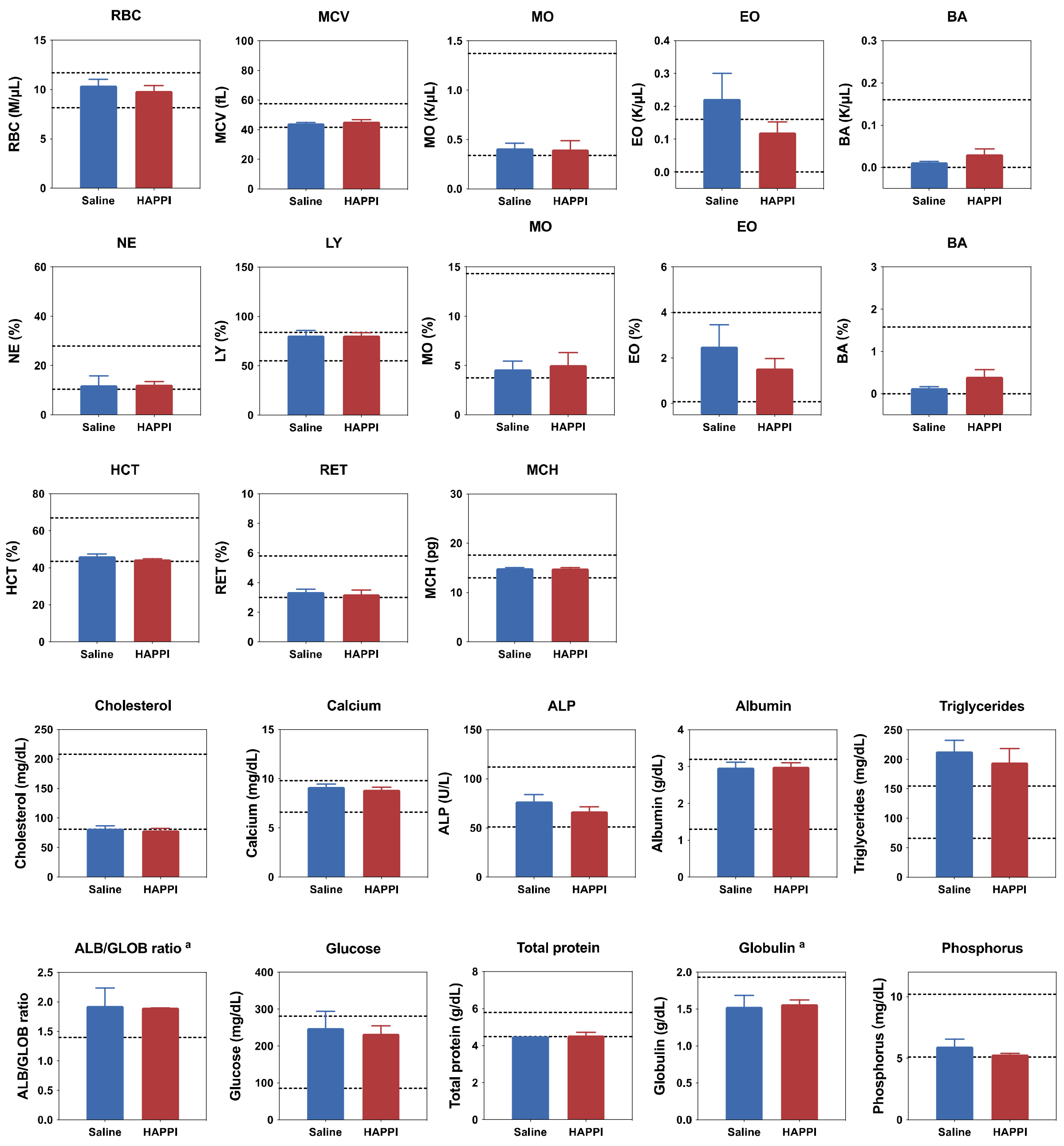

Fig. S3. Hematology and serum chemistry analysis. HAPPI was repeatedly dosed on Days 0, 30, 60 and 90 and blood was withdrawn at Day 97. Ab: RBC: red blood cells, MCH: mean corpuscular hemoglobin, MO: monocytes, EO: eosinophils, BE: basophils, MCV: mean corpuscular volume, HCT: hematocrit, RET: reticulocyte, ALP: alkaline phosphatase, ALB/GLOB ratio: albumin/globulin ratio. All data are mean ± SD. *n* = 3. Dashed lines indicate established normal ranges (two lines) or values (one line). [41–43]
